# Assessing Genomic Admixture between Cryptic *Plutella* Moth Species following Secondary Contact

**DOI:** 10.1093/gbe/evy224

**Published:** 2018-10-13

**Authors:** Christopher M Ward, Simon W Baxter

**Affiliations:** Department of Molecular and Biomedical Science, School of Biological Sciences, University of Adelaide, Australia

**Keywords:** introgression, hybridization, admixture, cryptic species, *Plutella xylostella*, *Plutella australiana*

## Abstract

Cryptic species are genetically distinct taxa without obvious variation in morphology and are occasionally discovered using molecular or sequence data sets of populations previously thought to be a single species. The world-wide Brassica pest, *Plutella xylostella* (diamondback moth), has been a problematic insect in Australia since 1882, yet a morphologically cryptic species with apparent endemism (*P. australiana*) was only recognized in 2013. *Plutella xylostella* and *P. australiana* are able to hybridize under laboratory conditions, and it was unknown whether introgression of adaptive traits could occur in the field to improve fitness and potentially increase pressure on agriculture. Phylogenetic reconstruction of 29 nuclear genomes confirmed *P. xylostella* and *P. australiana* are divergent, and molecular dating with 13 mitochondrial genes estimated a common *Plutella* ancestor 1.96 ± 0.175 Ma. Sympatric Australian populations and allopatric Hawaiian *P. xylostella* populations were used to test whether neutral or adaptive introgression had occurred between the two Australian species. We used three approaches to test for genomic admixture in empirical and simulated data sets including 1) the f3 statistic at the level of the population, 2) pairwise comparisons of Nei’s absolute genetic divergence (*d*_XY_) between populations, and 3) changes in phylogenetic branch lengths between individuals across 50-kb genomic windows. These complementary approaches all supported reproductive isolation of the *Plutella* species in Australia, despite their ability to hybridize. Finally, we highlight the most divergent genomic regions between the two cryptic *Plutella* species and find they contain genes involved with processes including digestion, detoxification, and DNA binding.

## Introduction

Cryptic species lack conspicuous variation in visible traits, yet can show high levels of ecological, behavioral, and genetic divergence, particularly when they arise in allopatry (Stuart et al. 2006; Bickford et al. 2007; Pfenninger and Schwenk 2007). Morphological resemblance of two or more distinct species can occur when environmental pressures maintain phenotypes or cause convergence, and through introgression of traits by interspecies hybridization (Bickford et al. 2007). Consequently, cryptic species are often overlooked, leading to both underestimates of species richness and overestimates of their geographic range (Stuart et al. 2006; Vodă et al. 2015).

Reproductive barriers can maintain boundaries between sympatric congeneric animal species (cryptic or noncryptic) using a range of isolating mechanisms such as olfaction, pheromone cues, and mating calls (Jones and Hamilton 1998; Andersson et al. 2007), host plant preference or mating timing (Hänniger et al. 2017), and endosymbiont infection (Shoemaker et al. 1999; Bordenstein et al. 2001). Although these factors can impose reproductive isolation barriers and restrict hybridization, assortative mating does not always occur (Mallet et al. 2007). Interspecific hybridization of two species within the same genera has been found to occur at similar rates across the animal kingdom, after taxonomic groups are adjusted for species richness (Schwenk et al. 2008). While hybridization between related species has been well documented, the process of distinguishing between adaptive introgression and regions of historic population structure has been challenging (Martin et al. 2015).

Closely related allopatric or sympatric species without gene flow should exhibit genetic divergence across the genome, whereas species with gene flow should show lower levels of divergence across broad regions relative to the frequency of interbreeding and how recently it occurred. Detecting hybridization is possible through the use of informal statistical tests on genetic variation, including principle component analysis (Patterson et al. 2006) and Bayesian STRUCTURE model analysis (Pritchard et al. 2000). While these tests can provide results indicative of admixture, they cannot distinguish between introgression, interlineage sorting, or homoplasic genetic drift. Patterson et al. (2012) formalized statistical approaches to estimate admixture based on allele frequencies across multiple populations, namely the f3 and f4 statistics (*D-*statistic), which assess the likelihood of hybridization. The f4 statistic has identified introgression between sympatric *Heliconius* butterfly species (Martin et al. 2013; Zhang et al. 2016) and hominids (Patterson et al. 2012), as allele frequencies across these genomes did not always agree with the expected species tree, or neutral drift.

Hybridization and introgression of genetic variation from a donor species into a recipient can have adaptive advantages. The transfer of advantageous preadapted alleles from one species into another removes the reliance of new traits arising though mutation in the recipient. Examples include the transfer of rodenticide resistance between mice (Song et al. 2011), coat color alleles among jackrabbits and hares (Jones et al. 2018), aposematic wing patterns in *Heliconius* butterflies (Mavárez et al. 2006; Pardo-Diaz et al. 2012; Wallbank et al. 2016) and insecticide resistance genes in *Anopholes* mosquitoes (Lee et al. 2013; Norris et al. 2015).

The diamondback moth, *Plutella xylostella* (L.) (Lepidoptera: Plutellidae), is the most destructive pest of Brassicaceous agricultural crops, including broccoli, cabbage, and canola (Furlong et al. 2013). They are able to cause *en masse* defoliation, malformed, and improper plant growth (Zalucki et al. 2012), and often develop resistance to insecticides making pest control an ongoing challenge. *Plutella xylostella* were first documented in Australia in the 1880s (Tyron 1889), yet an endemic and phenotypically cryptic species, *P. australiana* (Landry and Hebert), was only recently identified through high divergence of mitochondrial COI barcode sequences (8.6%) and morphologically distinct genitalia (Landry and Hebert 2013). The discovery was surprising, as *P. australiana* was not detected in previous molecular studies of *P. xylostella* yet, is dispersed across eastern Australia (Endersby et al. 2006; Delgado and Cook 2009).

Insecticide susceptibility appears to limit *P. australiana*’*s* pest potential among cultivated brassica crops, however, introgression of insecticide resistance loci from *P. xylostella* could have serious consequences for agriculture. *Plutella xylostella* and *P. australiana* can hybridize in experimental laboratory crosses, despite their contrasting infection rates of endosymbiotic *Wolbachia* (Ward and Baxter 2017; Perry et al. 2018), which are known to cause reproductive incompatibility in some cases (Sasaki and Ishikawa 2000; Duplouy et al. 2013). *Wolbachia* infection is fixed among *P. australiana* yet extremely low in Australian *P. xylostella* (1.5%). Although the strength of reproductive barriers in the field is unknown, limited numbers of SNP markers widely dispersed across the nuclear genome previously identified genetic structure between sympatric populations of *P. xylostella* and *P. australiana* (Perry et al. 2018). Due to *P. australiana*’s apparent endemism and the relatively recent invasion of *P. xylostella* into Australia, we assessed the capacity for sympatric Australian *Plutella* species to exchange beneficial traits through disassortative mating and introgression in the field through analyzing whole genomes.

## Materials and Methods

### Specimen Collection and Genome Sequencing

*Plutella xylostella* and *P. australiana* were collected from canola (*Brassica napus*) fields using light traps at Cook, Australian Capital Territory (ACT), (−35.262, 149.058) in October 2014 and from direct larval sampling at Ginninderra Farm, ACT, (−35.187, 149.053) in December 2015. Larvae from Calca, South Australia, (SA) (−33.049, 134.373) and Bairds Bay, (SA) (−33.023, 134.279) were collected in June 2014 from mixed stands of sand rocket (*Diplotaxis tenuifolia*) and wall rocket (*D. muralis*). Larval collections were reared through to pupation then frozen, to eliminate samples infected with parasitoids. A single *P. australiana* moth was also collected from Richmond, New South Wales (−33.597, 15.740) using a light trap. Large populations of *P. xylostella* larvae were also collected from *Brassica* vegetable farms on three Hawaiian Islands in August 2013, including Kunia, Oahu (21.465, −158.064), Kula, Maui (20.791, −156.337) and Waimea on Hawaii Island (20.028, −155.636), and reared for one generation. Genomic DNA purification was performed using phenol extractions, treated with RNaseA, precipitated with ethanol, and resuspended in TE buffer (10 mM Tris, 0.1 mM EDTA). Species identification was performed using a PCR-RFLP diagnostic assay of the mitochondrial COI gene (Perry et al. 2018). Genome sequencing was performed using the Illumina HiSeq2500 or NextSeq platforms at the Australian Genome Research Facility and the Australian Cancer Research Facility.

### Processing Genome Sequence Data

Summary statistics of Illumina sequence reads were generated with FastQC (Andrews 2010) and visualized using the R package ngsReports (Ward et al. 2018). Trimmomatic v 0.32 (Bolger et al. 2014) was used with the parameters (TRAILING: 15 SLIDINGWINDOW: 4: 15) to trim adapter, quality filter, and retain paired reads. The *P. xylostella* reference genome (You et al. 2013) was downloaded from NCBI (GCA_000330985.1). Stampy v1.0.21 (Lunter and Goodson 2011) was used to align the paired reads to the reference with the parameters (*–gatkcigarworkaround, –substitutionrate = 0.01*) which produced Sequence Alignment/Map (SAM) files that were converted to binary format (BAM) and indexed then sorted using SAMtools v1.2 (Li et al. 2009). PCR and optical duplicates were removed using Picard Tools v1.61 (http://broadinstitute.github.io/picard/). BAM summary statistics including average read depth per site called, coverage of the genome, percent missing data, total number of reads and read quality were generated using SAMtools v1.2.

### Genotype Variant Calling

Variant calling was performed using the Genome Analysis ToolKit (GATK) v3.3 (DePristo et al. 2011). GATK: HaplotypeCaller was used to generate gVCF records, containing variant and invariant sites across the genome, on a per sample basis. The HaplotypeCaller parameter heterozygosity (likelihood of a site being nonreference) for each species was estimated by SAMtools v1.2, indicating *P. xylostella* from Hawaii was most similar to the reference genome (heterozygosity: *P. australiana* = 0.0497; *P. xylostella* Australia = 0.0348; *P. xylostella* Hawaii = 0.0272). Individual gVCF records were combined using GATK: Genotype GVCF and filtered using BCFtools (Li et al. 2009) to a minimum individual depth greater than five reads per base with no greater than 40% of samples missing genotypes at any one site.

Nei’s mean intrapopulation nucleotide diversity, π, (Nei and Li 1979) was calculated using egglib (De Mita and Siol 2012). The mean and standard error in π and jackknifing was performed using the R package bootstrap (Canty and Ripley 2017). Pairwise *F*_ST_ and Tajima’s *D* was calculated across 50-kb windows using VCFtools (Danecek et al. 2011) and minimum distances between populations (km’s) determined with http://www.movable-type.co.uk/scripts/latlong.html, last accessed October 25, 2018.

### Phylogenetic Reconstruction of *Plutella* Mitochondrial and Nuclear Genomes

All quality filtered variant and invariant sites called against the mitochondrial reference genome (GenBank KM023645) were extracted using BCFtools and converted to a FASTA alignment using the R programming language. Maximum likelihood phylogenetic inference using the nuclear genome consensus, heterozygous sites were replaced with IUPAC ambiguity codes, alignment was performed with exaML (Kozlov et al. 2015) with GTR+GAMMA bootstrap resampling (*n* = 100; GTR+GAMMA) was then carried out using RAxML v8.2.4 to provide node confidence. The phylogeny was then rooted using the midpoint method in FigTree (v1.4.3, http://tree.bio.ed.ac.uk/software/figtree).

### De Novo Assembly of Mitochondrial Genomes and *Plutella* Split Time Estimates

De novo assembly of *Plutella* mitochondrial genomes was performed using NOVOPlasty v2.6.3 (Dierckxsens et al. 2017). A sequence read that mapped to the *P. xylostella* mitochondrial COI gene was used as the seed to initiate assembly. Genomes circularized by NOVOPlasty were then annotated through homology to the *P. xylostella* mitochondrial reference gene annotation (GenBank KM023645) with Geneious v10.0.6. Potential misassemblies were investigated by mapping individual raw reads to the appropriate de novo assembly on a per sample basis using BWA-MEM (Li 2013). Mapped reads were then used as fragments in Pilon (Walker et al. 2014) to correct the assembly. The sample with the greatest total length (15,962 bp), *Paus ACT14.1*, was used to produce a reference for the mitochondrial genome of *Plutella australiana* (Genbank accession MG787473.1).

The mitochondrial split time between *P. xylostella* and *P. australiana* was estimated using 13 mitochondrial protein coding genes extracted from 20 *Plutella* samples with circularized genomes plus *Prays oleae* (accession no. NC_025948.1) and *Leucoptera malifoliella* (accession no. JN790955.1). Nucleotide alignments were made for each gene using MAFFT (Katoh et al. 2002), substitution models were determined using JModelTest2 (Darriba et al. 2012) and alignments were then imported into BEAUTi (Drummond et al. 2012). We set the clock model to strict with 0.0177 substitutions Myr^−1^ according to Papadopoulou et al. (2010). Substitution models were unlinked to allow each sequence to coalesce independently with the Yule speciation model. MCMC sampling was carried out over 1000000 trees sampling every 1000 using BEAST2 v 2.4.7 (Bouckaert et al. 2014). Sampled trees from the chain were checked using Tracer v 1.6 (Rambaut et al. 2018) to determine burn in. Densitree was then used to superimpose MCMC trees to determine the internal node height ranges.

### Data Simulation

Coalescent local trees with a total chromosomal length of 25 Mb were simulated for 24 individuals, including eight samples from an outgroup (O) and two ingroups (*I*_1_ and *I*_2_) using the Markovian Coalescent Simulator, MaCS (Chen et al. 2009). A coalescent model for the most recent common ancestor of *I*_1_ and *I*_2_ was set to 0.4 × 4 *N* generations ago and the root to 1.5 × 4 N generations ago, providing the topology ((*I*_1_, *I*_2_), O). Simulated divergence was determined using mean *d*_XY_ values from *Plutella* samples (see fig. 4). Two approaches were used to simulate introgression events from *I*_2_ to O or from O to *I*_2_. First, Introgression was simulated as a single *en masse* admixture event at 0.01 × 4 N generations ago with admixture frequencies (*f*) of *f = *0, 0.05, 0.1, 0.2, and 0.3. Second, introgression was simulated over five distinct breakdowns in assortative mating (0.01, 0.008, 0.006, 0.004, and 0.002 x 4 N generations ago) and *f = *0, 0.05, 0.1, 0.2, and 0.3. Each simulation was carried out with a constant population recombination rate (4Nr) of 0.001. Sequences were generated from the coalescent trees using SeqGen (Rambaut and Grassly 1997) with the Hasegawa–Kishino–Yano substitution model (Hasegawa et al. 1985) and a branch scaling factor of 0.01.

### Admixture

#### The F3-Statistic

A formal test for admixture was calculated using the three population test, the f-statistic (f3) (Reich et al. 2009; Patterson et al. 2012). Three possible combinations of tip structures were assessed with the ingroups and outgroup namely f3(*I*_1_, *I*_2_; O), f3(*I*_1_, O; *I*_2_), f3(*I*_2_, O; *I*_1_,). Cases without introgression are expected to return positive f3 values while negative values indicate introgression has occurred from a donor to a recipient population, forming an intermediate ancestor of both source populations. Block jack-knife F3 estimation was carried out using PopStats (https://github.com/pontussk/popstats).

#### Absolute Divergence (d_XY_)

Nei’s absolute divergence, *d*_XY_, was used to calculate the mean number of nucleotide differences between two populations across nonoverlapping 50-kb windows with *egglib_sliding_windows.py* (https://github.com/johnomics). Comparisons of *d*_XY_ were made first with simulated data sets, using the five admixture frequencies (*f *=* *0, 0.05, 0.1, 0.2, and 0.3) between *I*_2_ and O, O and *I*_2_ and *I*_1_ and *I*_2_. The *d*_XY_ values were summarized by transforming them into density plots to visualize the distribution and frequency across the simulated genome. This provided expected *d*_XY_ patterns under a range of admixture frequencies. Average *d*_XY_ was then calculated between 1) *P. australiana* (O) and Australian *P. xylostella* (*I*_2_) individuals, 2) *P. australiana* (O) and Hawaiian *P. xylostella* (*I*_1_) and 3) Hawaiian *P. xylostella* (*I*_1_) and Australian *P. xylostella* (*I*_2_). Histograms were plotted after setting the maximum value to 1 using the *geom_density* function in ggplot2 (Wickham 2009).

#### Tree-Tip Distance Proportions

Maximum likelihood phylogenetic reconstruction was performed with RAxML (Stamatakis 2014) using nonoverlapping 50-kb genomic windows generated with the python script *genoToSeq.py* (https://github.com/simonhmartin). Phylogenies of empirical data each used four individuals, including one *P. xylostella* and one *P. australiana* individual from sympatric Australia populations (SA14, ACT14, or ACT15), and two *P. xylostella* individuals from Hawaii (HO13.1 and HH13.2). Each tree was then converted to a distance matrix using APE (Paradis et al. 2004) and pairwise distances between tips were determined in the R programming language using the equation,

$$equation\;1\;\frac{d_{a}}{{d_{a}+\;d}_{b}}=\;{Proportion}_{ab}$$

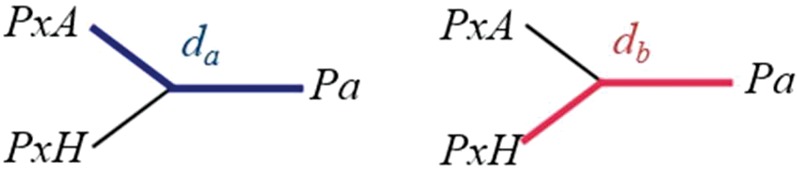
where *d*_a_ is the branch distance between the tree-tip of an Australian *P. xylostella* individual (*PxA*) and a *P. australiana* individual (*Pa*) and *d*_b_ is the averaged branch distance between two Hawaiian *P. xylostella* (*Pxyl HO13.1* and *Pxyl HH13.2*) and a *P. australiana* individual*.* Two individuals from Hawaii were used to reduce bias from this ingroup source. The *Proportion*_ab_ values for each 50-kb window were expected to be ∼0.5 if the phylogeny was concordant with the species tree. Values much >0.5 indicate genomic windows more similar between *P. australiana* and Hawaiian *P. xylostella*, while values much <0.5 indicate genomic windows more similar between *P. australiana* and Australian *P. xylostella* and are candidate admixed regions. Only genomic windows with >20% of sites genotyped were analyzed.

For comparison, simulated data from 24 individuals and five admixture frequencies (*f *=* *0, 0.05, 0.1, 0.2, and 0.3) described earlier was divided into 50-kb windows (*n* = 500). Each 50-kb window was then subdivided into 64 separate alignments containing four simulated samples; the same two *I*_1_ individuals in each case, (reflecting the use of the same two *P. xylostella* samples from Hawaii in the empirical data) and nonredundant pairs of *I*_2_ and O individuals. Four tip unrooted maximum likelihood phylogenies were produced for each alignment using RAxML, then Proportion_ab_ calculated and plotted using a bin width of 0.05.

### Analysis of Discordant Tree-Tip Distances

After plotting tree-tip distance proportions, the tails of each distribution was investigated for symmetry by counting the number of 50-kb windows above or below each mean at three thresholds (mean ± 0.05, 0.10, and 0.15). Windows below the mean (mean – 0.15) were further investigated by calculating *d*_XY_ across 10-kb windows, sliding by 2 kb. These genomic regions indicate greater similarity between *P. australiana* and Australian *P. xylostella* than the average and *d*_XY_ plots were visually inspected for signs of introgression.

### Identification of Divergent Genomic Windows between *P. australiana* and *P. xylostella*

Both F_ST_*and d*_XY_ were calculated across aligned 50-kb genomic windows between all *P. xylostella* samples (from Australia plus Hawaii) and *P. australiana.* Annotated protein coding genes were extracted from the most divergent 1% of 50-kb windows for each statistic and BLAST against the DBM gene list available from DBM-DB (Tang et al. 2014). To identify their molecular function, InterPro and UniProt annotations were obtained for each BLAST hit.

## Results

### Alignment of *Plutella* Species to the Reference Genome

The genomes of 29 *Plutella* samples were sequenced using short read Illumina platforms, including eight *P. xylostella* from Hawaii, eight *P. xylostella* from Australia, and 13 *P. australiana*. Samples from Australia were classified into three populations based on collection location and year for analysis (ACT2014, ACT2015, SA2014). A single *P. australiana* individual from Richmond, NSW, was also sequenced (supplementary table S1, Supplementary Material online). Resequenced genomes were mapped to the ∼393 Mb *P. xylostella* reference genome (You et al. 2013), but just 170 Mb of non-N bases were retained after stringent quality filtering. Sequence coverage across the 170 Mb alignment ranged from 9- to 25-fold per individual and ∼70% of these sites were genotyped in *P. australiana* samples compared with ∼92% for Australian and Hawaiian populations of *P. xylostella* (table 1).

**Table 1:** Summary of Sequence Coverage, Percentage of Sites Genotyped, and Mean Nucleotide Diversity of *Plutella* Populations (170 Mb)

Population	Year Collected	Species	Number of Samples	Average Coverage Per Site	Sites Genotyped (%)	Mean Nucleotide Diversity	(±SD)
Australian Capital Territory (ACT)	2014	*P. xylostella*	2	17	91.5	0.0151	(0.0042)
		*P. australiana*	4	12.5	71.8	0.0170	(0.0052)
Australian Capital Territory (ACT)	2015	*P. xylostella*	2	18	92.2	0.0150	(0.0045)
		*P. australiana*	4	13.5	72.3	0.0168	(0.0048)
South Australia (SA)	2014	*P. xylostella*	4	23.5	91.8	0.0157	(0.0040)
		*P. australiana*	4	17.5	65.9	0.0174	(0.0051)
Hawaii	2013	*P. xylostella*	8	13.125	91.7	0.0200	(0.0044)

The highest levels of nucleotide diversity were observed within Hawaiian *P. xylostella* samples (table 1). However, endemic *P. australiana* populations showed higher levels of nucleotide diversity than Australian *P. xylostella*, which may have undergone a population genetic bottleneck when colonization occurred. Mutation-drift equilibrium of these populations was determined using Tajima’s *D* (*D*_T_). *Plutellaxylostella* collected from Australia were under equilibrium (*D*_T_ 95% CI = −0.6046375 to +0.9435148) whereas those collected from Hawaii showed largely negative values (*D*_T_ 95% CI = −1.88 to −0.039) which may be the result of a recent population size expansion or higher than expected abundance of rare alleles. The frequency of rare alleles in *P. australiana* was also common, although the *D*_T_ 95% confidence interval overlapped with zero (*D*_T_ 95% CI = −1.18 to + 0.42).

Pairwise comparisons between populations and species were then used to assess genetic structure with *F*_ST_. The three Australian *P. xylostella* populations showed no genetic structure between geographic location (SA vs. ACT) or year (2014 vs. 2015) (combined average of *F*_ST_ =0.003 ± 0.003), as has been previously reported with microsatellite data (Endersby et al. 2006). However, much higher levels of differentiation were observed when compared with Hawaiian *P. xylostella*, supporting the expectation of genetic isolation (average of *F*_ST_ =0.108 ± 0.01). The average pairwise *F*_ST_ values were slightly lower between *P. australiana* and Hawaiian *P. xylostella* (*F*_ST_ =0.501 ± 0.002) than *P. australiana* and Australian *P. xylostella* (*F*_ST_ =0.532 ± 0.013) (table 2).

**Table 2:** Matrix of the Minimum Distance between Collection Sites (km’s, Above Diagonal) and Pairwise *F*_ST_ Values of Each *Plutella* Population (below diagonal)

Species and Population	*Pxyl*.ACT.2014	*Pxyl*.ACT.2015	*Pxyl*.SA.2014	*Pxyl*.Hawaii.2013	*Paus*.ACT.2014	*Paus*.ACT.2015	*Paus*.SA.2014
*Pxyl*.ACT.2014		8.3	1372	8470	0	8.3	1372
*Pxyl*.ACT.2015	0.000		1371	8466	8.3	0	1371
*Pxyl*.SA.2014	0.007	0.006		9480	1372	1371	0
*Pxyl*.Hawaii.2013	0.102	0.103	0.119		8470	8466	9480
*Paus*.ACT.2014	0.521	0.520	0.548	0.499		8.3	0
*Paus*.ACT.2015	0.523	0.523	0.549	0.500	0.001		8.3
*Paus*.SA.2014	0.525	0.525	0.551	0.503	0.003	0.008	

### Phylogenetic Inference of *Plutella* Species

**Fig. 1.**
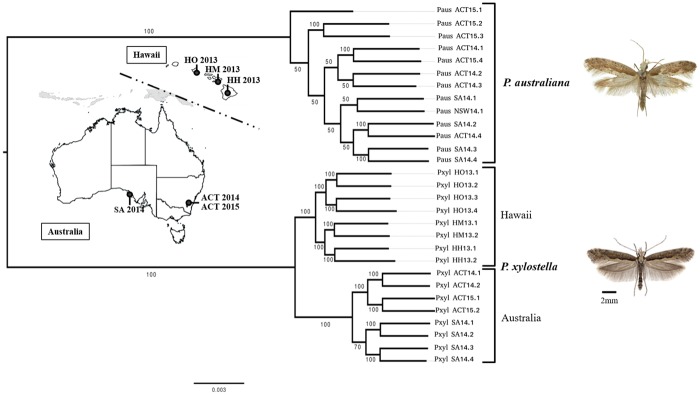
—Maximum likelihood phylogeny of *Plutella xylostella* and *P. australiana* generated using a 170-Mb concatenated alignment of the nuclear genome. Bootstrap support (*n* = 100) is shown at each node. The inner maps show population locations and year collected for samples from Australia (SA, South Australia; ACT, Australian Capital Territory) and Hawaii, USA (HO, Hawaii Oahu; HM, Hawaii Maui; HH, Hawaii, Hawaii Island). See table 2 for distances between collection locations. Insect photographs were provided by Paul Hebert (*P. australiana*) and Jean-François Landry (*P. xylostella*).

A maximum likelihood phylogeny using ∼170 Mb of the nuclear genome showed two clear *Plutella* species groups with deep divergence between species. *Plutella xylostella* from Hawaii and Australia formed reciprocally monophyletic sister clades with 100% bootstrap support while *P. australiana* genomes formed a single clade, although generally had lower levels of internal branch support (fig. 1). Branch distances were shorter between the internal nodes of *P. australiana* and Hawaiian *P. xylostella* than Australian *P. xylostella*, suggesting the two *P. xylostella* clades have diverged substantially since their most recent common ancestor.

### *Plutella a* *ustraliana* Mitochondrial Genome and Dating

**Fig. 2.**
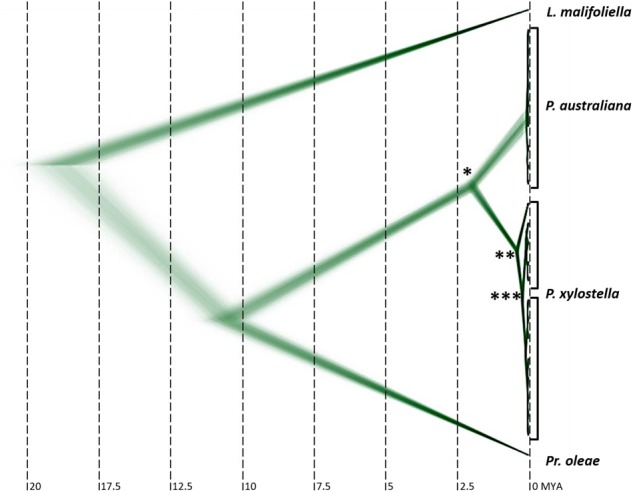
—Superimposed MCMC trees of 13 protein coding mitochondrial genes used to estimate the split time of *Plutella xylostella* (*n* = 13) and *P. australiana* (*n* = 9) at 1.96 ± 0.175 Ma (*). The internal node of the *P. xylostella* clade was estimated at 0.37 ± 0.057 Ma (**). The split of Hawaiian and Australian *P. xylostella* haplotypes was estimated at 0.078 ± 0.024 Ma (***). *Prays oleae* (accession no. NC_025948.1) and *Leicoptera malifoliella* (accession no. JN790955.1) were used as outgroups.

We carried out de novo assembly and annotation of the *P. australiana* mitochondrial genome which has a total length of 15,962 bp (GenBank accession MG787473.1) compared with 16,014 bp of *P. xylostella* (Dai, Zhu, Qian, et al. 2016). Using sequence homology to the *P. xylostella* mitochondrial genome we annotated two rRNAs, 13 protein coding mitochondrial genes and 22 t-RNA, which showed a conserved gene order for Lepidopteran mitochondrial genomes (Dai, Zhu, Zhao, et al. 2016). The nucleotide sequence of 13 protein coding mitochondrial genes from 22 *Plutella* samples were then used to estimate the mitochondrial split time between *P. xylostella* and *P. australiana* at 1.96 Ma (95% confidence interval ± 0.175 Myr, fig. 2 and supplementary table S2, Supplementary Material online). *Prays oleae* and *Leicoptera malifoliella* were used as the outgroups. The topology of the 13 mitochondrial genes used to date the split (supplementary table S3, Supplementary Material online) also supported two clear *Plutella* species groups with an average of 4.95% divergence. *Plutella xylostella* from Hawaii showed higher mitochondrial diversity than samples from Australia. Reduced mitochondrial diversity may have been caused by a founder effect when Australia was colonized (Perry et al. 2018).

### Assessing Admixture between Australian *Plutella* Species

#### The F3-Statistic

**Fig. 3.**
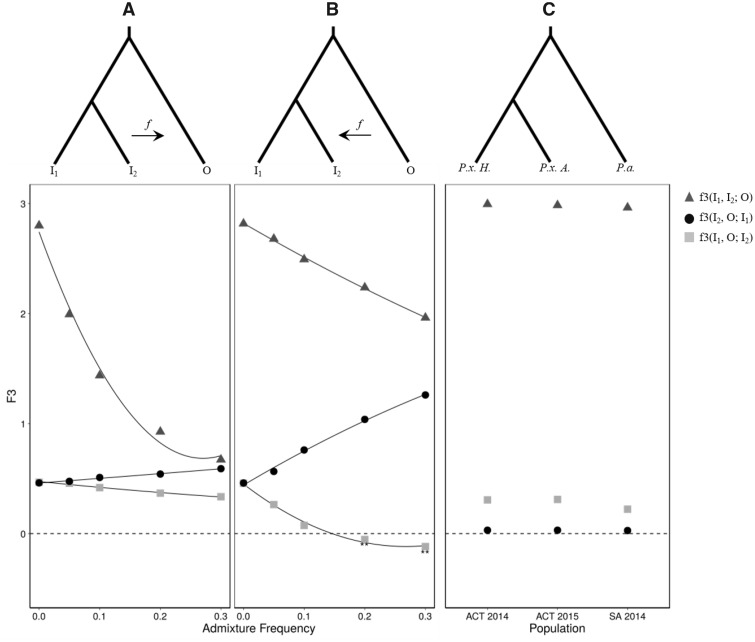
—The three population f-statistic (f3). (*A*) Admixture from ingroup 2 (*I*_2_) to the outgroup (O) was simulated as a single event with frequencies of *f *=* *0, 0.05, 0.1, 0.2, and 0.3. Evidence for hybridization and admixture could not be clearly detected in this direction, as shown by the gray boxes for f3(*I*_1_, O; *I*_2_), which did not reach negative values. For comparison, the f3-statistic for (*I*_2_, O; *I*_1_) and (*I*_1_, *I*_2_; O) were plotted with circles and triangles, respectively. (*B*) Simulated admixture from O into *I*_2_ did produced a significant f3 statistic at a mixing frequency >0.2, as indicated by the values <0 f3(*I*_1_, O; *I*_2_). (*C*) The f3-statistic was then applied to empirical data, testing for admixture in three possible scenarios between *Plutella xylostella* from Hawaii (*P.x. H, I*_1_), *P. xylostella* from Australia (*P.x. A, I*_2_) and *P. australiana* (*P.a.*, O). This suggests that, as no f3 values were <0, if admixture was occurring it could not be detected using this method.

A formal test for genomic admixture was calculated using the three-population f-statistic (f3), first with simulated data sets to assess the level of sensitivity we could reasonably achieve, and second with empirical data. Simulated introgression frequencies of *f = *0.0, 0.05, 0.1, 0.2, and 0.3 were applied from a donor to a recipient. Introgression from ingroup 2 (*I*_2_) into the outgroup (O) increased similarity between these groups, yet also reduced genetic differences between the outgroup and ingroup 1 (*I*_1_). Despite the outgroup becoming more similar to *I*_2_, O still contained a large proportion of divergent loci which tends to confound the f3 statistic making negativity difficult to achieve, even with high levels of introgression (Peter 2016). Consequently, this approach failed to indicate shared ancestry through a negative f3-statistic (fig. 3*A*). Next, introgression from O into *I*_2_ was simulated, to assess sensitivity of introgression from *P. australiana* into Australian *P. xylostella*. Negative values were detected for mixing frequencies of ≥20% (*f *=* *0.2), indicating high rates of recent hybridization are required to detect introgression using the f3-statistic (fig. 3*B*). Interestingly, spreading the total proportion of introgression to five equidistant time-points along the branch did not increase the detectability of admixture (supplementary fig. S1**,**Supplementary Material online). This suggests the f3 is more dependent on the admixture frequency than the divergence between discordant and concordant regions.

Applying the f3-statistic to empirical data failed to identify negativity in any tip order between Australian *P. xylostella* and *P. australiana* (fig. 3*C*). Results for the f3-statistic were lower when assessing introgression between Hawaiian *P. xylostella* and *P. australiana* than from between the two sympatric Australian species, consistent with the nuclear phylogeny showing Hawaiian samples are more similar to *P. australiana*. A lower f3(*Pxyl* Hawaii, *Pau.*; *Pxyl* Australia) value was estimated for SA 2014 than ACT 2014 and ACT 2015, which may be due to differences in nucleotide diversity between the Australian populations (table 1), as f3 is decreased proportional to the frequency of minor alleles in the target population. As f3 did not detect recent admixture events with two closely related ingroup tips, further tests were used to investigate introgression using smaller genomic windows.

#### Absolute Divergence (d_XY_)

Nei’s measure of absolute divergence (*d*_XY_) (Nei 1987) was used to compare genetic similarity between populations using 50-kb genomic windows for both simulated and empirical data sets. In all cases, population wide comparisons of *d*_XY_ were performed between; 1) two ingroup populations (*I*_1_ and *I*_2_), 2) ingroup 1 and the outgroup (*I*_1_ and O), and 3) ingroup 2 and the outgroup (*I*_2_ and O). Comparions returing values approaching zero indicate high levels of similarity and a recent allelic split time. Low *d*_XY_ values are expected between ingroup samples (*I*_1_ and *I*_2_), or in cases where introgression may be occurring between an ingroup and outgorup.

**Fig. 4.**
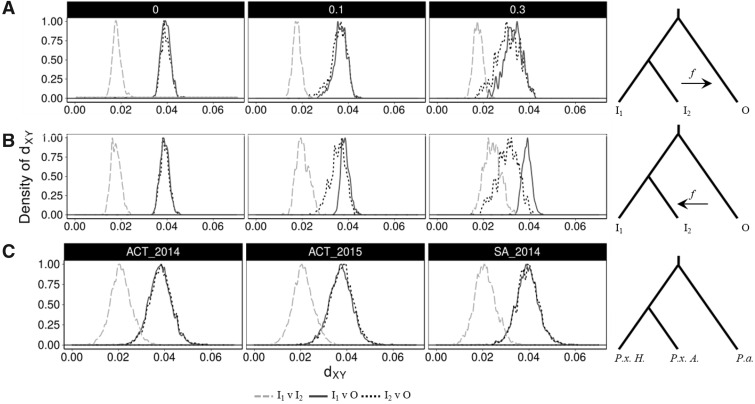
—Absolute genetic divergence (*d*_XY_) between populations. Each plot is a histogram summarizing pairwise comparisons of 50-kb windows across the genome, rescaled such that the maxima is 1. Simulated data uses mixing frequencies of *f *=* *0.0, 0.1, and 0.3 (see supplementary figs. S2 and S3**,**Supplementary Material online, for additional admixture frequencies) (*A*) Simulated *d*_XY_ comparisons assessing of admixture from *I*_2_ into O or (*B*) O into *I*_2_. In the abseence of hybridization (*f = *0) the simulated ingroups show the lowest levels of divergence (dashed line), while the distance between each ingroup and the outgroup are relatively similar (dotted and solid lines). Increasing levels of admixture (*f *=* *0.1, 0.3) alters histogram shape as *I*_2_ and O *d*_XY_ values become smaller. (*C*) *d*_XY_ summaries between *Plutella australiana* (*P.a.* in the phylogeny schematic) and *P. xylostella* from Australia (*P.x. A*) or Hawaii (*P.x. H*) do not deviate. This indicates *d*_XY_ was not able to detect hybridization at the population level.

Absolute divergence in simulated populations was calculated for each 50-kb window (*n* = 500), again for admixture occuring at *f *=* *0.0, 0.05, 0.1, 0.2, and 0.3. The distribution of *d*_XY_ values obtained for each comparison were plotted as histograms normalized for density by rescaling such that the maxima of the distribution is 1 (fig. 4 andsupplementary figs. S2 and S3, Supplementary Material online). Introgression either from *I*_2_ into O or from O into *I*_2_ produced a decrease in absolute divergence across the genome, providing a benchmark for comparisons with empirical data. Admixture in the direction O to *I*_2_ provided a much clearer genome wide signal than the reverse direction, (*I*_2_ to O) indicating it would be easier to detect introgression from *P. australiana* into Australian *P. xylostella* than the reverse.

Based on the whole genome phylogeny (fig. 1), we expected mean *d*_XY_ between *P. australiana* and Hawaiian *P. xylostella* to be slightly lower than Australian *P. xylostella*. The *d*_XY_ distribution of empirical 50-kb windowed data provided no support of widespread introgression (fig. 4*C*), as values comparing the *P. australiana* outgroup with either *P. xylsotella* from Hawaii (*I*_1_) or Australia (*I*_2_) did not deviate from their expected values (table 3). This suggests concordance with the whole genome tree topology.

**Table 3:** Confidence Intervals (95%) for *d*_XY_ Comparisons of Populations

	*Pxyl.* Hawaii vs*. Pxyl.* Australia	*Pxyl.* Hawaii vs*. Paus.* Australia	*Pxyl.* Australia vs*. Paus.* Australia
ACT 2014	0.02101–0.02122	0.03788–0.03812	0.03828–0.03852
ACT 2015	0.02102–0.02123	0.03721–0.03744	0.03771–0.03795
SA 2014	0.02087–0.02111	0.03898–0.03926	0.03926–0.03955

#### Phylogenetic Tree-Tip Distance Proportions

The f3-statistic and *d*_XY_ were both used to test for introgression within populations. Next, we used the tree-tip distance proportion to assess whether evidence for introgression could be detected between individual sample pairs. Simulated genomes with introgression from *I*_2_ into O, or O into *I*_2_ at the rates *f *=* *0.0, 0.05, 0.1, 0.2, and 0.3 were divided into 50-kb sequence alignments, as described earlier. Further subdivision was then performed so each 50-kb window contained just four sequences; two ingroup 1, one ingroup 2, and one outgroup sequence. This was repeated 64 times for each 50-kb window, then maximum likelihood phylogenic reconstruction performed for each alignment. Based on the whole-genome topology, we expected the outgroup to be a similar distance from both ingroup 1 and ingroup 2, unless introgression had occurred and shortened the distance between samples.

**Fig. 5.**
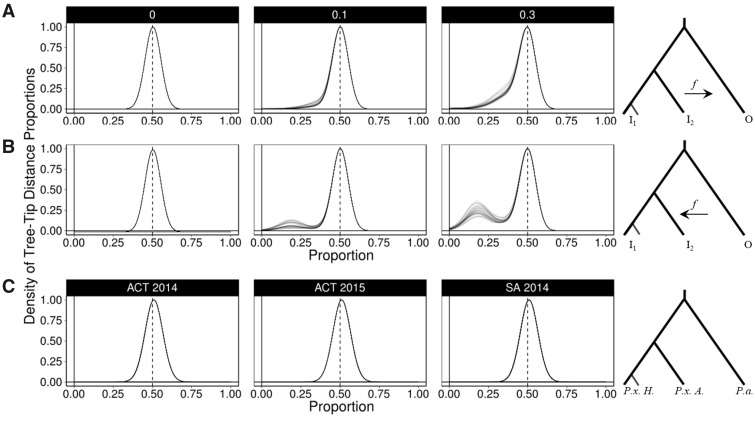
—Histogram summaries of tree-tip distance proportions, depicting the phylogenetic distance between ingroup and outgroup sequences. A ratio of 0.5 indicates the outgroup sample has the same branch distance to both ingroup samples. Ratios close to zero indicate very short branch lengths between ingroup two and the outgroup, and are candidate regions for introgression. (*A*) Simulated introgression from ingroup two to the outgroup at mixing frequencies of *f = *0, 0.1 and 0.3. (*B*) Simulated introgression from the outgroup into ingroup two at mixing frequencies of *f = *0, 0.1 and 0.3. (*C*) Empirical data for the three sympatric Australian *Plutella* populations (ACT 2014, ACT 2014, SA 2014). Each panel summarizes 7276 (±293) branch distance ratio calculations. The branch leading to ingoup 1 was standardized using the same two Hawaiian *P. xylostella* individuals for each of these comparisons.

A proportion of the branch distance between *I*_2_ and O (*d*_a_) and *I*_1_ and O (*d*_b_) was then calculated for each phylogeny using equation 1, normalizing values within the range 0–1. Tree-tip distance proportions are presented as histograms to graph the distribution (*x axis*), and normalized density (*y axis*). Although introgression from *I*_2_ into O (fig. 5*A* and supplementary fig. S4**,**Supplementary Material online) and from O into *I*_2_ (fig. 5*B*) were both detected using distance proportions, patterns did vary based on the direction of admixture. Clearer signals of admixture were evident in the direction O to *I*_2_ (supplementary table S4**,**Supplementary Material online), as this made ingroup 2 less similar to ingroup 1. Given *I*_1_ and *I*_2_ recently split, admixture from *I*_2_ into O is also expected to make the outgroup more similar to *I*_1_, decreasing detectability. Simulating introgression over five equaly spaced events was effective at detecting admixture using tree-tip distance proportions (supplementary fig. S5**,**Supplementary Material online).

This method was then applied to empirical data to identify genomic windows that were discordant with the species tree. Similar to the simulated data sets, genomic alignments were divided into nonoverlapping 50-kb contiguous windows, then further subdivided into alignments of one *P. xylostella* and one *P. australiana* individual from sympatric Australian populations, plus two consistant *P. xylostella* from Hawaii. This produced 32 different sample combinations for each 50-kb window, including eight combinations from ACT 2014, eight from ACT 2015 and 16 from SA 2014. An average of 7276 (±293) maximum likelihood phylogenies were then produced for each of the 32 sample combinations to identify potential admixture that was not fixed in the population. A near-symetric and unimodal distribution of tree-tip distance proportions was observed in all cases, with the ranges of each density curve showing a large degree of overlap (fig. 5*C* and supplementary table S5**,**Supplementary Material online). Mean tree-tip distance proportions for each comparison were consistant within and between populations ACT 2014 (0.5088–0.5104), ACT 2015 (0.5102–0.5117), and SA 2014 (0.5086–0.5099). All proportion means were >0.5 showing the Hawaiian *P. xylostella* had on average shorter branch lengths to *P. australiana*, consistant with the nuclear genome phylogeny (fig. 1). Under a widespread admixture hypothesis windows with distance proportions below the mean (*P. australiana* closer to *P. xylostella* from Australia) should be much more frequent than above. The number of windows above and below three distances from the mean (0.05, 0.1, 0.15) was similar (supplementary table S6**,**Supplementary Material online) suggesting no clear evidence to support widespread admixture within *P. xylostella* and *P. australiana* individuals from three sympatric populations.

Despite lack of support for widespread hybridization and genome-wide introgression, we further investigated the tails of the tree-tip distance proportions at a distance 0.15 below the mean for each Australian population. These scaffolds (*n* = 21) have the shortest branch lengths between *P. australiana* and sympatric Australian *P. xylostella*, relative to the branch length proprtions between *P. australiana* and Hawaiian *P. xylostella* (supplementary table S7**,**Supplementary Material online). Sliding window *d*_XY_ was performed on each of these scaffolds with 10-kb windows (sliding by 2 kb), revealing just one region on scaffold KB207303.1 where Australian *P. xylostella* and *P. australiana* are more similar than between Hawaiian and Australian *P. xylostella*. Historical admixture between Australian *Plutella* species is one possible explanation for this result, although the region does not contain any protein coding genes (supplementary fig. S6*A***,**Supplementary Material online). A region on scaffold, KB207380.1, was identfied in the tree-tip distribition tail in 12/32 comparisons however *d*_XY_ indicated admixture across this region was unlikely (supplementary fig. S6*B***,**Supplementary Material online).

### Genomic Regions with High Interspecies Divergence

The two *Plutella* species investigated in this study have been shown to have contrasting biologies and pest potential (Perry et al. 2018), and although they can hybridize in laboratory crosses, we found no evidence for widespread admixutre among wild samples. This prompted us to ask which 50-kb genomic regions are most divergent between these species, and what kinds of genes do they encode? First, absolute divergence (*d*_XY_) between all *P. xylostella* and all *P. australiana* individuals was used to identify the top 1% most divergent genomic windows (supplementary table S8**,**Supplementary Material online). These included fifty-one 50-kb windows dispersed across 41 unique scaffolds and showed 33–61% greater absolute divergence than the genome-wide average (*d*_XY_=0.0369). Second, the top 1% of genomic windows showing highest divergence in nucleotide diversity (*F*_ST_) were also identified, showing values 70–110% higher than the mean (*F*_ST_=0.356). These two estimates of divergence only detected one 50-kb region common to both *d*_XY_ and *F*_ST_ (KB207411.1; 400,001…450,000 bp). Most windows with the highest *d*_XY_ had relatively low *F*_ST_ values, suggesting the two species share similar levels of polymorphism across these regions. Nonredundant protein coding genes (*n* = 176) within these divergent genomic windows contained genes required for feeding including digestion (eg. chymotrypsin, trypsin, aminopeptidase-N), detoxification (eg. cytochrome P450s, carboxylesterases) and also gene regulation (zinc finger proteins) (supplementary table S9**,**Supplementary Material online). However, Tajima’s *D* showed most of these windows were within the genome wide 95% confidence intervals, indicating these regions are not likely to be under directional or balancing selection (supplementary figs. S7 and S8, Supplementary Material online).

## Discussion

The discovery of cryptic species can often be inadvertent and arise from sequencing mitochondrial or nuclear amplicons (Stuart et al. 2006; Landry and Hebert 2013), as well as whole genomes (Janzen et al. 2017). The fortuitous identification of *Plutella australiana* was unexpected and raised initial concern over its pest status and whether specific management practices were required. *Plutellaaustraliana* populations collected from across southern Australia (Perry et al. 2018) and Australian *P. xylostella* (Endersby et al. 2006) lack genetic structure, showing these species are highly mobile. Adaptive introgression of advantageous traits from one of these species into the other could potentially spread across the Australian continent. Despite high levels of movement, we sampled from sites where *Plutella* populations co-occur to attempt to detect either historical admixture or very recent hybridization.

The physical genome size of *P. xylostella* is estimated at 339 Mb (Baxter 2011) while the reference genome assembly is 393 Mb (You et al. 2013) and includes sequencing gaps totaling ∼50 Mb. After aligning all resequenced genomes to the *P. xylostella* reference, only 170 Mb was retained in this analysis, which is likely to be caused in part by the sequence gaps and also high levels of genetic diversity (You et al. 2013). Mapping *P. australiana* sequence reads to the *P. xylostella* genome is affected by mapping bias, as the most divergent loci will not map to this reference. This causes all branches to be shortend toward the reference, underestimating the diveregence between *P. australiana* and *P. xylostella* in the whole genome phylogeny. However, the introduction of this bias is unavoidable as the only reference geneome within the superfamily Yponomeutoidea is currenlty *Plutella xylostella.*

*Plutella australiana* were more similar to *P. xylostella* samples from Hawaii than Australia, based on shorter phylogenetic branch lengths for nuclear genomes and subsequent tree-tip distance proportions, lower *F*_ST_ values and lower f3-statistics. A better understanding of migration or transport routes enabling *P. xylostella* to colonize the world would help explain why this is the case. Several studies have found Australian *P. xylostella* mtDNA genomes have very low levels of diversity (Saw et al. 2006; Juric et al. 2017; Perry et al. 2018), which is indicative of a population bottleneck (and other factors), while we found Hawaiian mtDNA genomes to be quite diverse. This suggests the Hawaiian Islands may have been colonized by a larger founding population, or multiple, independent invasions while Australia may have simply been colonized by a derived population of *P. xylostella* (Juric et al. 2017).

Mitochondrial diversity between the two *Plutella* species was originally found to be ∼8.2%, based on sequencing COI amplicons (Landry and Hebert 2013), although the level of diversity across all thirteen protein coding genes is less (4.95%). This level of diversity was not sufficient to result in complete reproductive isolation between the two sister species when reared in the laboratory (Perry et al. 2018). Using the 13 mitochondrial genes, we estimated the split time of *P. xylostella* and *P. australiana* to be ∼1.96 Myr. To date, *P. australiana* has only been detected in Australia, yet this relatively recent split questions whether *P. australiana* did evolve within Australia. This would require a considerable migratory event some 2 Mya from the ancestral *Plutella* source population to Australia, and no further migration. Future molecular screening of *P. xylostella* may identify cryptic *P. australiana* in other countries.

Phylogenies of genes or genomic windows can deviatie from an expected consensus topology or species tree and can be used to identify genomic regions that may be of biological interst. For example, genomic regions subject to incomplete lineage sorting (Scally et al. 2012), horizontal gene transfer (Moran and Jarvik 2010) and adaptive introgression (Wallbank et al. 2016) all produce discordant phylogenies. Despite simulated data detecteting minor levels of introgression using phylogenetic tree-tip distances across the genome, we found few discordant distances between individual *Plutella* samples across 50-kb genomic windows. The methods used here were not sufficient to reject small regions of decreased *d*_XY_ between Australian *Plutella*, which may be signals of past admixture. Future work into the evolutionary history of *Plutella* moths and sequencing outgroup genomes of *Plutella* species, will enable further analysis of these regions using the *D* statistic and *f*_d_ (Martin et al. 2015).

The most divergent genomic windows between the two *Plutella* species identified using *d*_XY_ or *F*_ST_ showed little evidence for current selection and may potentially contain genes that underwent selection after speciation. These genes may reflect different abilities to evade host plant defenses or host plant preference, as many are involved with digestion and detoxification. Using absolute genetic divergence (*d*_XY_) to identify the most divergent genomic windows between *P. australiana* and *P. xylostella* may also be highlighting loci that are highly polymorphic or rapidly evolving. Further understanding of *Plutella* biology including mating timing, evolutionary history, host plant preference and behavious may provide further insight into these divergent loci.

*Plutella australiana* and *P. xylostella* are likely to have been in secondary contact in Australia for over 125 years (>1000 generations). Despite this, we found no support for widespread admixture, and although we cannot predict the amount of time these species have spent in geographic isolation, strong reproductive barriers are apparent in the field. Furthermore, *P. xylostella* and *P. australiana* will be a useful system to investigate the genetic basis of biological differences between cryptic species from an agricultural perspective. 

## Supplementary Material

Supplementary Data
