# Supplementary material for: Assessing Genomic Admixture between Cryptic Plutella Moth Species following Secondary Contact
Source: Genome Biol Evol. 2018 Oct 13;10(11):2973–85. doi: 10.1093/gbe/evy224 (PMC6250210; doi:10.1093/gbe/evy224)
Supplement: Supplementary Data [file evy224_supp.zip › WardBaxter_SupplementaryFigures_Revision.docx]

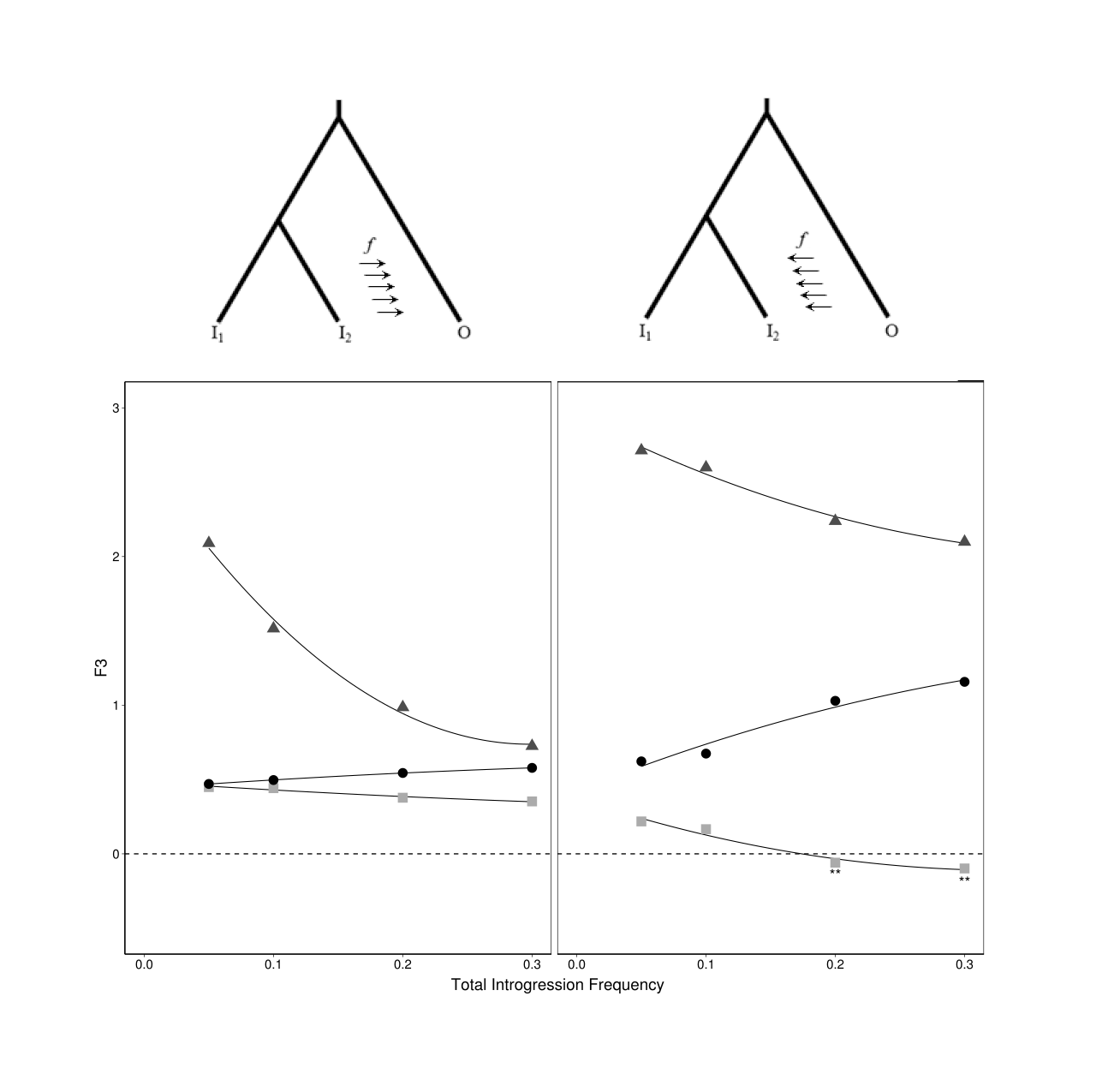


**Figure S1**: The f3 statistic was calculated on simulated data following introgression at five distinct time points along the branch (0.01, 0.008, 0.006, 0.004 and 0.002 x 4N generations ago). Four different introgression frequencies (*f*) were analysed including 0.01*f* x 5 time points = 0.05, *f =* 0.02 x 5 = 0.1, *f =* 0.04 x 5 = 0.2 and 0.06 x 5 = 0.3. The F3 statistic was then calculated for each admixture frequency for introgression simulated in the direction I_2_🡪O (left panel) and O🡪I_2_ (right panel). The f3 values generated from five simulated introgression events were similar to f3 values generated from single introgression events, shown in Figure 3.


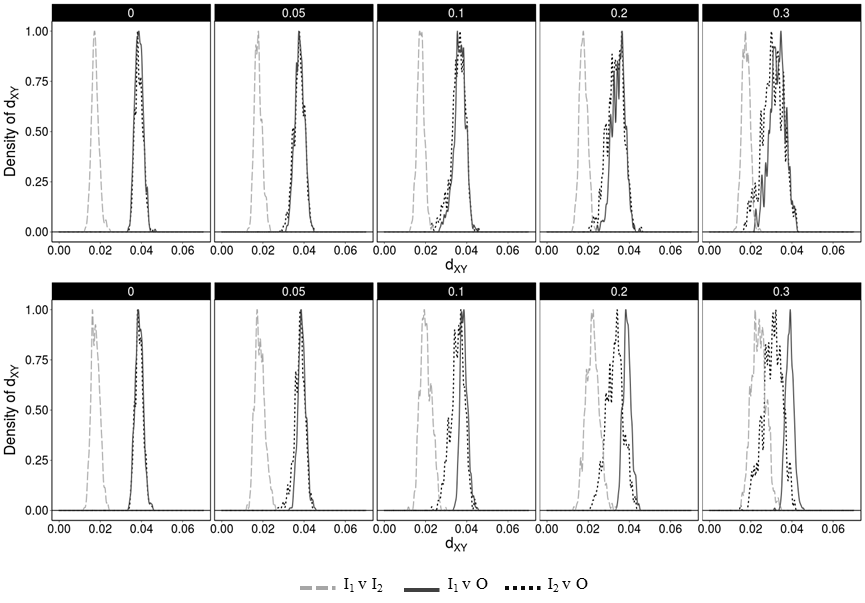
**Figure S2**: Density plots of absolute genetic divergence (*d_XY_*) following a single, unidirectional admixture event between simulated populations. Admixture was simulated at a range of frequencies (*f* = 0, 0.05, 0.1, 0.2, 0.3) from I_2_ to O (upper panel) or O into I_2_ (lower panel). See **Figure 4** for additional information.


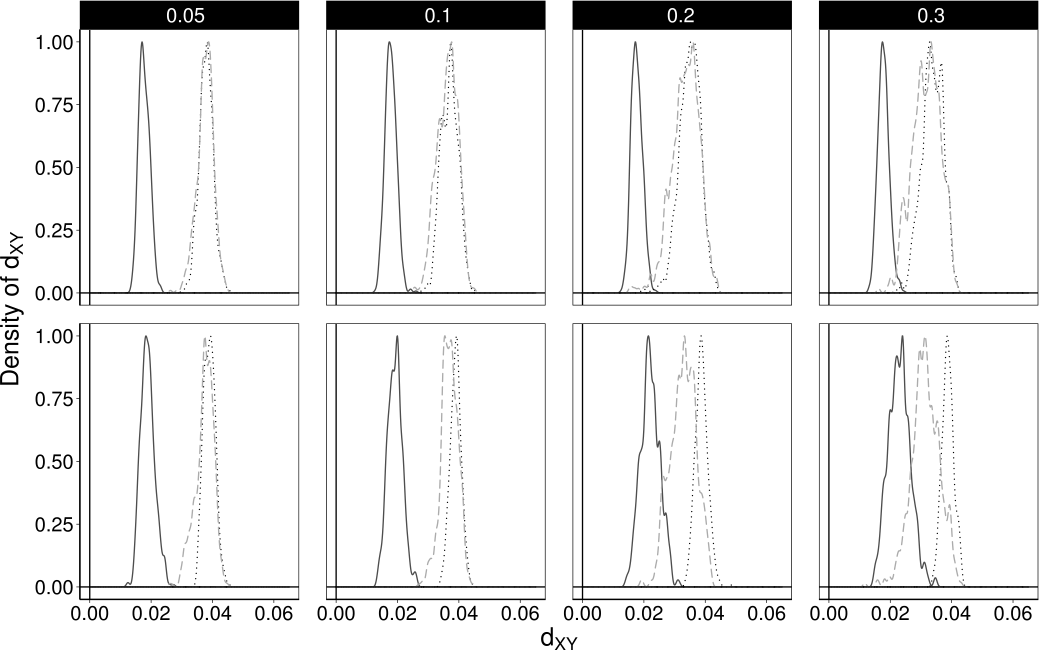


**Figure S3**: Density plots of absolute genetic divergence (*d_XY_*) following five, unidirectional admixture event along the branch between simulated populations. Four different introgression frequencies (*f*) were analysed including 0.01*f* x 5 time points = 0.05, *f =* 0.02 x 5 = 0.1, *f =* 0.04 x 5 = 0.2 and 0.06 x 5 = 0.3 from I_2_🡪O (upper panel) or O🡪I_2_ (lower panel).


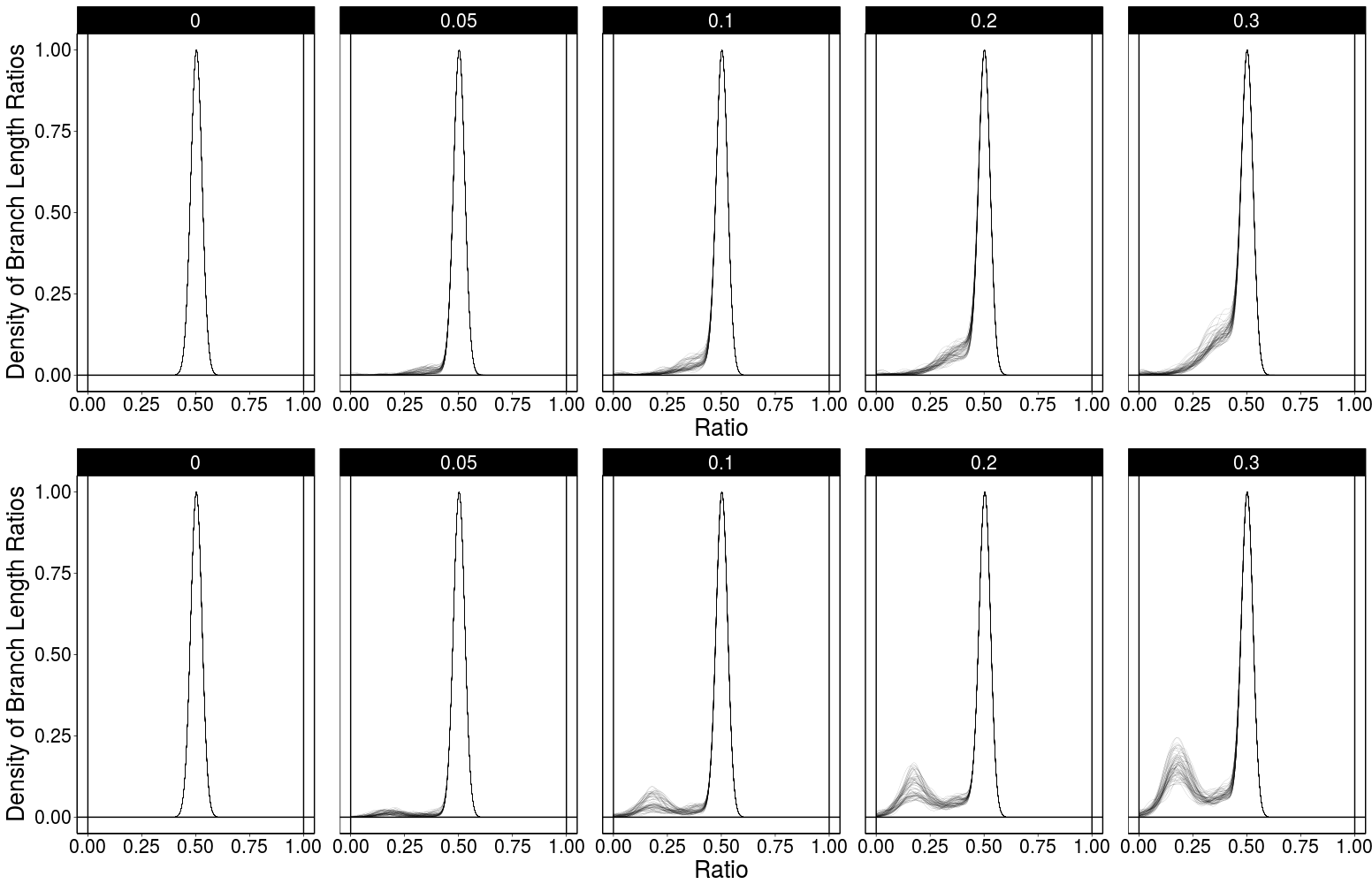


**Figure S4**: Histogram densities of tree-tip distance proportions, depicting the phylogenetic distance between simulated ingroup and outgroup sequences. Introgression was simulated from I_2_ to O (upper panel) or from O into I_2_ (lower panel) using a single unidirectional admixture event at frequencies of *f* = 0, 0.05, 0.1, 0.2, 0.3. Refer to **Figure 5** of the main text for further description and Table S4 for 95% confidence intervals.


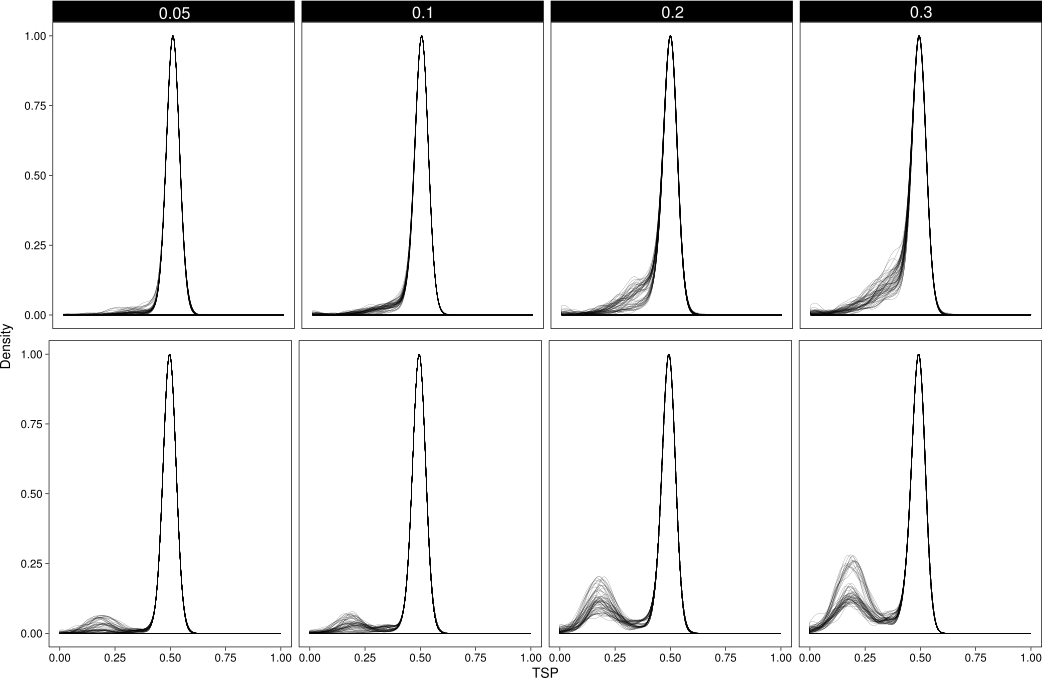


**Figure S5**: Histogram densities of tree-tip distance proportions, depicting the phylogenetic distance between simulated ingroup and outgroup sequences. Introgression was simulated from I_2_ to O (upper panel) or from O into I_2_ (lower panel) using five distinct time points along the branch with admixture events at frequencies of, *f* = 0, 0.05, 0.1, 0.2, 0.3. Refer to **Figure 5** of the main text for further information.

**A.**


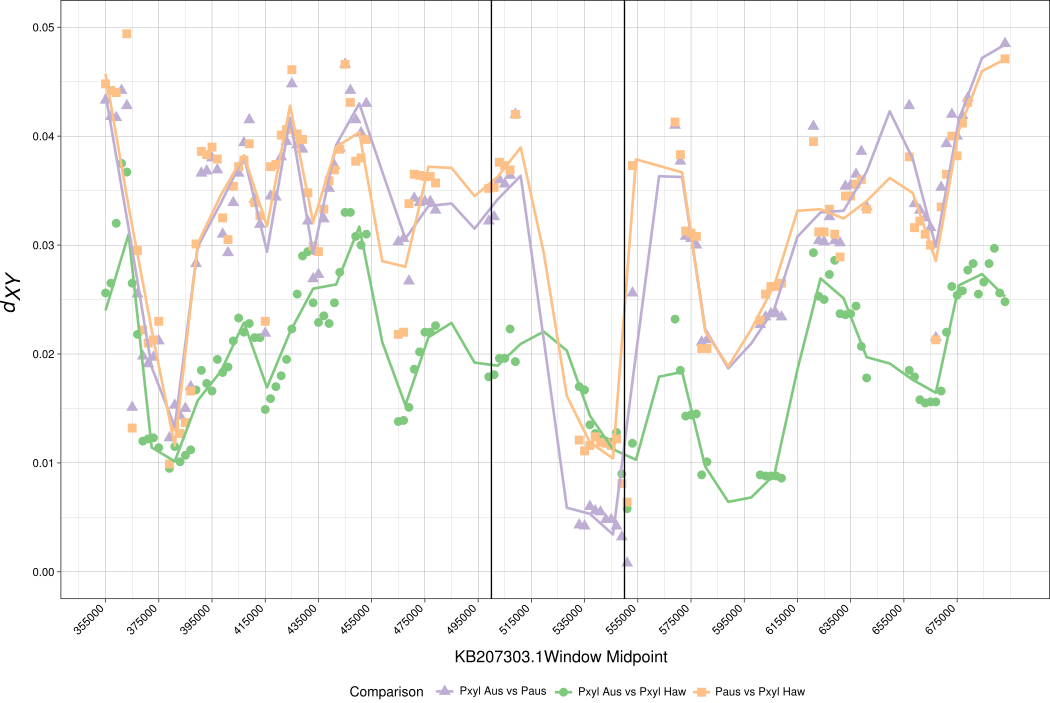


**B.**


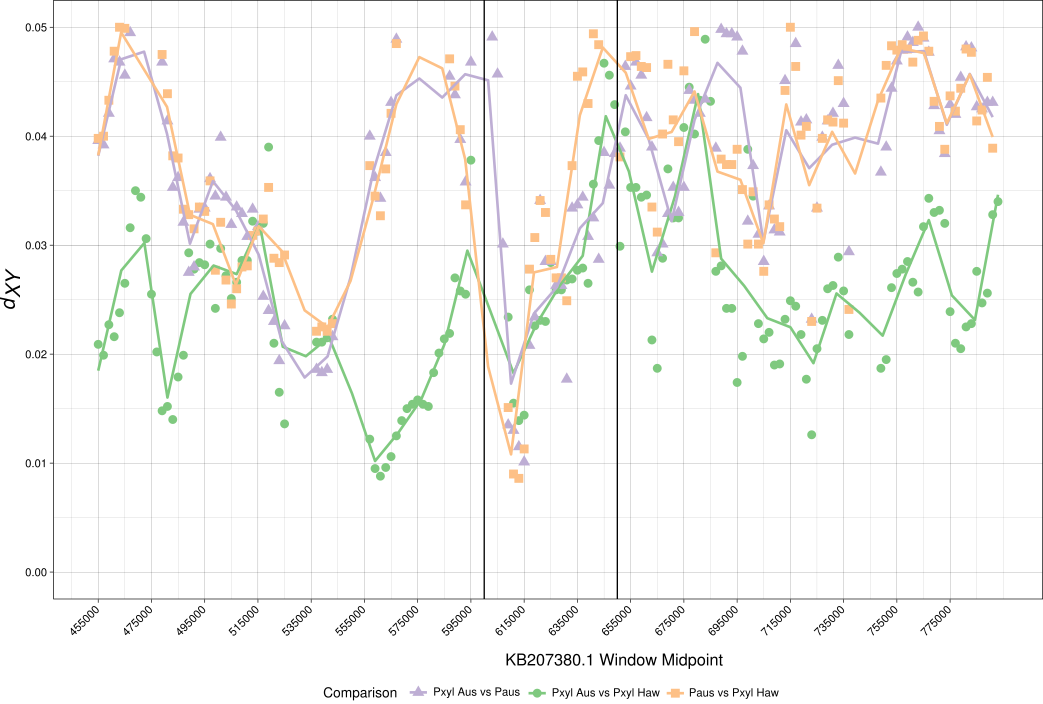


**Figure S6.** Absolute divergence (*d_XY_*) across two genomic scaffolds (10 kb windows, sliding by 2kb) comparing *P. australiana* and *P. xylostella* from Australia (purple), *P. australiana* and *P. xylostella* from Hawaii (orange) and *P. xylostella* from Hawaii and Australia (green). Tree-tip distance proportions indicated these two scaffolds (KB207303.1 and KB207380.1) had short branch lengths between Australian *P. xylostella* and *P. australiana* and were candidate regions for historical introgression. **A.** *Plutella australiana* and *P. xylostella* from Australia show the lowest *d_XY_* values around 525-550 kb of scaffold KB207303.1, providing some support for historical admixture. Vertical black lines highlight the 50 Kb window identified with the tree-tip distance proportion. **B.** Hawaiian *P. xylostella*  show high *d_XY_* when compared to both *P. australiana* (orange line) and Australian *P. xylostella* (green line) for scaffold KB207380.1 (620-660 Kb). Therefore, the tree-tip distance proportion identified this region (vertical black lines) due to increased divergence of the Hawaiian samples, opposed to increased similarity between Australian *P. xylostella* and *P*. *australiana*, and admixture is not supported.

**A.**


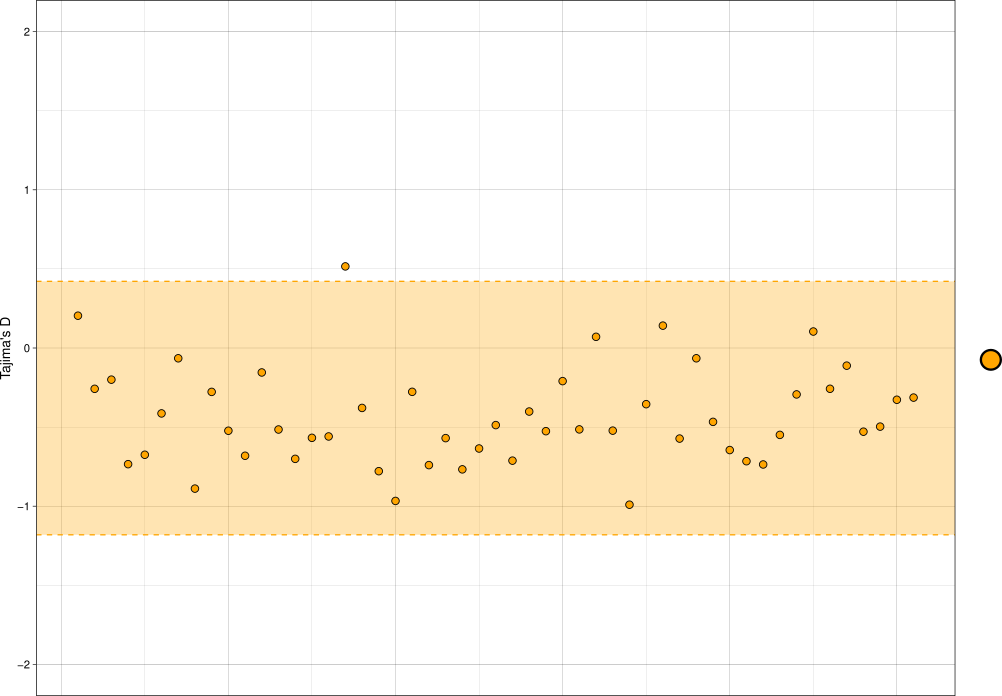


*P. australiana*


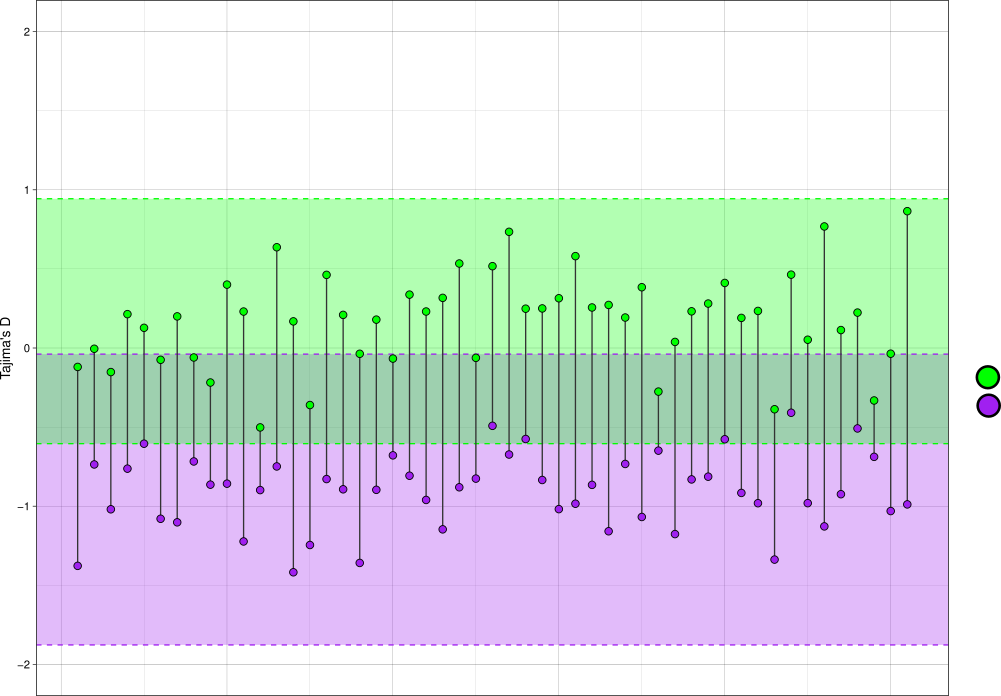


*P. xylostella* Australia

*P. xylostella* Hawaii

**B.**

*P. xylostella* Hawaii

*P. xylostella* Australia

*D_XY_* outlier window

**Figure S7:** Tajima’s D was calculated for each *Plutella* populations in 50kb windows across the nuclear genome. Tajima’s D for each 50 Kb *d_XY_* outlier windows is plotted in the same order as presented in Table S9. The 95% confidence intervals calculated across the genome are shaded. **A)** *P. australiana* (orange) values of Tajima’s D for each window. **B)** Tajima’s D for *P. xylostella* populations, Australia (green) and Hawaii (purple), linked with a vertical line for each window.

**A.**


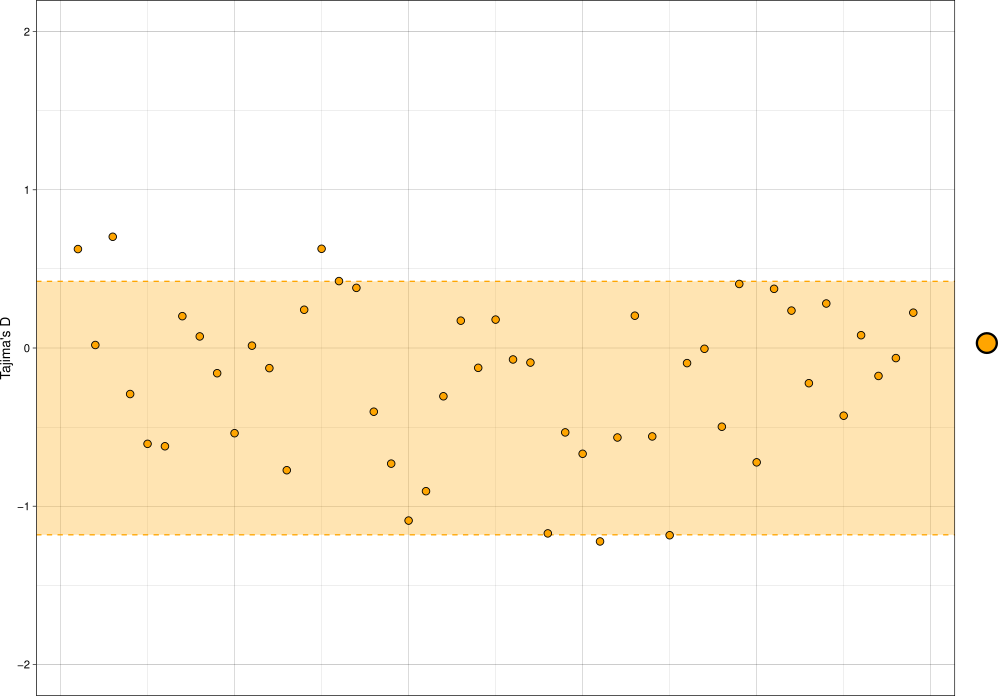


*P. australiana*


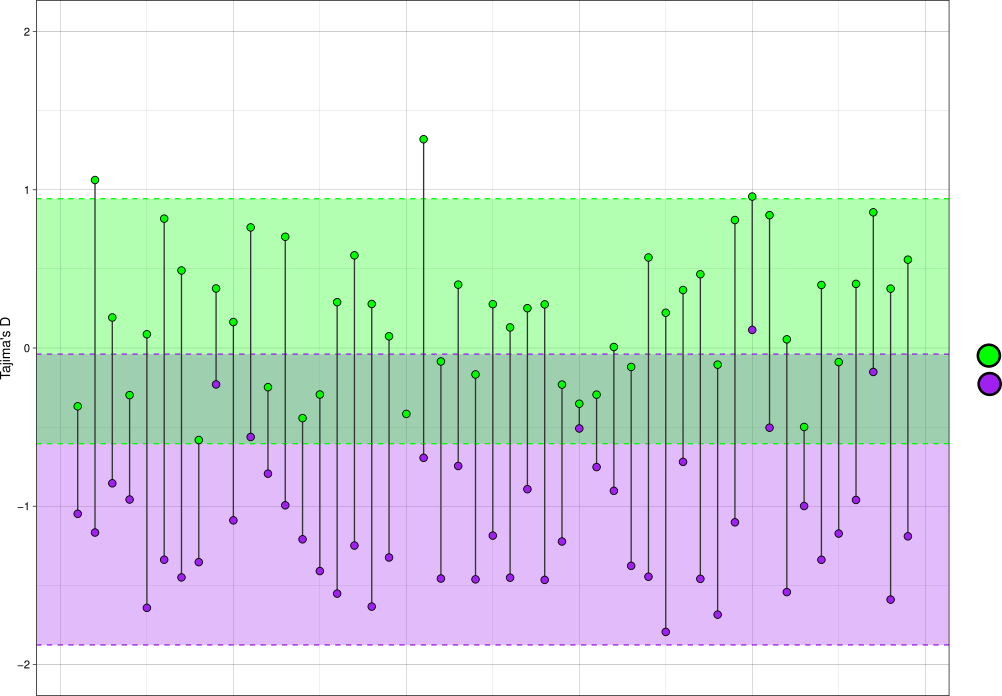


*P. xylostella* Australia

*P. xylostella* Hawaii

**B.**

F_ST_ outlier window

**Figure S8:** Tajima’s D was calculated for each *Plutella* populations in 50kb windows across the nuclear genome. Tajima’s D for each 50 kb *F_ST_* outlier windows is plotted in the same order as presented in Table S9. The 95% confidence intervals calculated across the genome are shaded. **A)** *P. australiana* (orange) values of Tajima’s D for each window. **B)** Tajima’s D for *P. xylostella* populations, Australia (green) and Hawaii (purple), for each window.
