# Supplementary material for: Assessing Genomic Admixture between Cryptic Plutella Moth Species following Secondary Contact
Source: Genome Biol Evol. 2018 Oct 13;10(11):2973–85. doi: 10.1093/gbe/evy224 (PMC6250210; doi:10.1093/gbe/evy224)
Supplement: Supplementary Data [file evy224_supp.zip › WardBaxter_SupplementaryTables_Revision.docx]

**Table S1.** *Plutella* species sample names, collection locations, sequence alignment summaries and accession numbers.

| **Population (Australia or Hawaii)** | **Sample name** | **Collection location** | **Latitude** | **Longitude** | **Aligned single reads (millions)** | **Read depth over 170 Mb of aligned data** | **SRA accession** |
| --- | --- | --- | --- | --- | --- | --- | --- |
| NSW 2014 | *PausNSW14.1* | *Richmond* | -33.597 | 150.740 | 39.63 | 9 | SRR6505278 |
|  |  |  |  |  |  |  |  |
| SA 2014 | *PausSA14.1* | *Bairds Bay* | -33.02288 | 134.27938 | 77.68 | 17 | SRR6505277 |
| SA 2014 | *PausSA14.2* | *Bairds Bay* | -33.02288 | 134.27938 | 68.07 | 15 | SRR6505279 |
| SA 2014 | *PausSA14.3* | *Calca* | -33.04916 | 134.37285 | 82.41 | 19 | SRR6023624 |
| SA 2014 | *PausSA14.4* | *Calca* | -33.04916 | 134.37285 | 84.74 | 19 | SRR6505269 |
| SA 2014 | *PxylSA14.1* | *Bairds Bay* | -33.02288 | 134.27938 | 93.64 | 23 | SRR6505223 |
| SA 2014 | *PxylSA14.2* | *Bairds Bay* | -33.02288 | 134.27938 | 91.29 | 22 | SRR6505222 |
| SA 2014 | *PxylSA14.3* | *Calca* | -33.04916 | 134.37285 | 97.50 | 24 | SRR6505219 |
| SA 2014 | *PxylSA14.4* | *Calca* | -33.04916 | 134.37285 | 104.00 | 25 | SRR6505218 |
|  |  |  |  |  |  |  |  |
| ACT 2014 | *PausACT14.1* | *Cook* | -35.262015 | 149.058586 | 41.23 | 13 | SRR6505273 |
| ACT 2014 | *PausACT14.2* | *Cook* | -35.262015 | 149.058586 | 38.18 | 12 | SRR6505275 |
| ACT 2014 | *PausACT14.3* | *Cook* | -35.262015 | 149.058586 | 44.91 | 14 | SRR6505276 |
| ACT 2014 | *PausACT14.4* | *Cook* | -35.262015 | 149.058586 | 48.27 | 11 | SRR6505268 |
| ACT 2014 | *PxylACT14.1* | *Cook* | -35.262015 | 149.058586 | 53.92 | 18 | SRR6505233 |
| ACT 2014 | *PxylACT14.2* | *Cook* | -35.262015 | 149.058586 | 49.17 | 16 | SRR6505232 |
|  |  |  |  |  |  |  |  |
| ACT 2015 | *PausACT15.1* | *Ginninderra* | -35.187115 | 149.052534 | 38.60 | 12 | SRR6505270 |
| ACT 2015 | *PausACT15.2* | *Ginninderra* | -35.187115 | 149.052534 | 45.48 | 15 | SRR6505271 |
| ACT 2015 | *PausACT15.3* | *Ginninderra* | -35.187115 | 149.052534 | 46.13 | 14 | SRR6505272 |
| ACT 2015 | *PausACT15.4* | *Ginninderra* | -35.187115 | 149.052534 | 43.55 | 13 | SRR6505274 |
| ACT 2015 | *PxylACT15.1* | *Ginninderra* | -35.187115 | 149.052534 | 54.00 | 18 | SRR6505221 |
| ACT 2015 | *PxylACT15.2* | *Ginninderra* | -35.187115 | 149.052534 | 55.81 | 18 | SRR6505220 |
|  |  |  |  |  |  |  |  |
| Hawaii 2013 | *PxylHH13.1* | *Hawaii Island* | 20.028 | -155.636 | 53.07 | 12 | SRR6505225 |
| Hawaii 2013 | *PxylHH13.2* | *Hawaii Island* | 20.028 | -155.636 | 56.62 | 13 | SRR6505224 |
| Hawaii 2013 | *PxylHO13.1* | *Oahu* | 21.465 | -158.064 | 57.60 | 14 | SRR6505231 |
| Hawaii 2013 | *PxylHO13.2* | *Oahu* | 21.465 | -158.064 | 56.22 | 14 | SRR6505230 |
| Hawaii 2013 | *PxylHO13.3* | *Oahu* | 21.465 | -158.064 | 53.32 | 13 | SRR6505229 |
| Hawaii 2013 | *PxylHO13.4* | *Oahu* | 21.465 | -158.064 | 56.53 | 14 | SRR6505228 |
| Hawaii 2013 | *PxylHM13.1* | *Maui* | 20.791 | -156.337 | 49.36 | 12 | SRR6505227 |
| Hawaii 2013 | *PxylHM13.2* | *Maui* | 20.791 | -156.337 | 55.02 | 13 | SRR6505226 |

**Table S2.** Samples with circularized *de novo* mitochondrial genome assemblies used to date the split time between *P. xylostella* and *P. australiana*. Genomes were annotated using homology to the *P. xylostella* mitochondrial reference genome and *Paus ACT14.1* has been submitted to Genbank (accession MG787473.1). Sequence alignments are available from the corresponding author upon request.

| Sample | Length | %GC |
| --- | --- | --- |
| *Paus ACT14.1* | 15962 | 19.3 |
| *Paus ACT14.3* | 15582 | 18.9 |
| *Pxyl ACT14.1* | 15664 | 18.8 |
| *Pxyl ACT14.2* | 15662 | 19.6 |
| *Paus ACT15.1* | 15677 | 19.6 |
| *Pxyl HH13.1* | 15516 | 18.9 |
| *Pxyl HH13.2* | 15573 | 18.9 |
| *Pxyl HM13.1* | 15647 | 18.9 |
| *Pxyl HM13.2* | 15608 | 19.6 |
| *Pxyl HO13.1* | 15603 | 18.8 |
| *Pxyl HO13.2* | 15580 | 18.9 |
| *Pxyl HO13.3* | 15666 | 18.9 |
| *Paus NSW13.1* | 15647 | 19.6 |
| *Paus SA14.1* | 15623 | 19.7 |
| *Paus SA14.2* | 15640 | 18.9 |
| *Paus SA14.3* | 15639 | 19.6 |
| *Paus SA14.4* | 15616 | 19.6 |
| *Pxyl SA14.1* | 15584 | 18.8 |
| *Pxyl SA14.3* | 15609 | 18.8 |
| *Pxyl SA14.4* | 15621 | 19.6 |

**Table S3**. Substitution models estimated with JModelTest2 for 13 mitochondrial genes used to date the *P. xylostella* and *P. australiana* split time.

| **Gene** | **Length (bp)** | **Average**  **Paus-Pxyl difference (%)** | **Substitution Model** | **α-shape^1^** | **Proportion Invariant^2^** |
| --- | --- | --- | --- | --- | --- |
| **ATP6** | 678 | 5 | TIM2+G | 0.336 | - |
| **ATP8** | 171 | 1.8 | HKY+I | - | 0.451 |
| **COX1** | 1531 | 6.2 | TIM2+I | - | 0.674 |
| **COX2** | 682 | 3.9 | TIM+I | - | 0.656 |
| **COX3** | 789 | 3.3 | TIM2+I | - | 0.484 |
| **CYTB** | 1162 | 5.3 | TIM2+I | - | 0.613 |
| **ND1** | 950 | 3.8 | K3PU+G | 0.680 | - |
| **ND2** | 1025 | 5.1 | HKY+G | 0.193 | - |
| **ND3** | 358 | 5.4 | HKY+G | 0.342 | - |
| **ND4** | 1355 | 5.2 | HKY+I | - | 0.586 |
| **ND4L** | 294 | 7.2 | HKY+G | 0.227 | - |
| **ND5** | 1751 | 5.7 | HKY+G | 0.159 | - |
| **ND6** | 546 | 6.4 | HKY+I | - | 0.453 |

^1^The α-shape represents the shape of the gamma distribution in substitution model +G

^2^Proportion invariant refers to invariant sites in the substitution model +I

**Table S4**. The 95% confidence intervals (CI) and standard error (SE) for mean tree-tip distance proportions of simulated datasets plotted in **Figure S4**. The direction of introgression and mixing frequencies are indicated.

| Direction of introgression | Mixing frequency (*f*) | 95% CI of the mean ingroup-outgroup branch distance ratio | 95% SE of the mean branch distance ratio |
| --- | --- | --- | --- |
| I_2_🡪O | 0 | 0.50329 -0.50335 | 0.0006 - 0.0006 |
| I_2_🡪O | 0.05 | 0.49653 - 0.49790 | 0.0022 - 0.0026 |
| I_2_🡪O | 0.1 | 0.48727 - 0.48916 | 0.0037 - 0.0040 |
| I_2_🡪O | 0.2 | 0.47377 - 0.47586 | 0.0049 - 0.0051 |
| I_2_🡪O | 0.3 | 0.46095 - 0.46346 | 0.0056 - 0.0059 |
| O🡪I_2_ | 0 | 0.50288 - 0.50297 | 0.0005 - 0.0005 |
| O🡪I_2_ | 0.05 | 0.49001 - 0.49101 | 0.0042 - 0.0046 |
| O🡪I_2_ | 0.1 | 0.46400 - 0.46914 | 0.0069 - 0.0074 |
| O🡪I_2_ | 0.2 | 0.43342 - 0.43899 | 0.0092 - 0.0095 |
| O🡪I_2_ | 0.3 | 0.40610 - 0.41093 | 0.0103 - 0.0105 |

**Table S5**. Mean range of tree-tip distance proportions comparing *P. australiana* and *P. xylostella* from Hawaii and Australia. The mean range is similar to simulated datasets without introgression.

| Population | Mean Range | Error Range (95%) |
| --- | --- | --- |
| ACT 2014 | 0.50889 - 0.51043 | 0.0005377 - 0.0005839 |
| ACT 2015 | 0.51024 - 0.51172 | 0.0005343 - 0.0005514 |
| SA 2014 | 0.50866 - 0.50999 | 0.0005474 - 0.0006147 |

**Table S6:** Number of windows in the distribution tails of tree-tip distances from figure 5C. For each comparison, the number of windows proportionally 0.05, 0.10 and 0.15 above or below the mean were counted. Numbers of windows above and below the mean for each category are similar, indicating widespread introgression is not supported.

| Comparison of Individuals | | 0.05 | | 0.10 | | 0.15 | |
| --- | --- | --- | --- | --- | --- | --- | --- |
| ***P. australiana*** | ***P. xylostella*** | **above** | **below** | **above** | **below** | **above** | **below** |
| *Paus ACT14.1* | *Pxyl ACT14.2* | 380 | 341 | 21 | 25 | 2 | 4 |
| *Paus ACT14.2* | *Pxyl ACT14.1* | 396 | 330 | 14 | 24 | 1 | 4 |
| *Paus ACT14.2* | *Pxyl ACT14.2* | 345 | 341 | 13 | 21 | 0 | 3 |
| *Paus ACT14.3* | *Pxyl ACT14.1* | 386 | 324 | 13 | 16 | 0 | 3 |
| *Paus ACT14.3* | *Pxyl ACT14.2* | 374 | 329 | 12 | 17 | 1 | 1 |
| *Paus ACT14.4* | *Pxyl ACT14.1* | 316 | 267 | 14 | 20 | 0 | 3 |
| *Paus ACT14.4* | *Pxyl ACT14.1* | 397 | 354 | 15 | 31 | 4 | 2 |
| *Paus ACT14.4* | *Pxyl ACT14.2* | 314 | 281 | 10 | 15 | 2 | 1 |
| *Paus ACT15.1* | *Pxyl ACT15.1* | 349 | 265 | 20 | 20 | 1 | 0 |
| *Paus ACT15.1* | *Pxyl ACT15.2* | 313 | 262 | 14 | 21 | 1 | 1 |
| *Paus ACT15.2* | *Pxyl ACT15.1* | 360 | 277 | 18 | 15 | 0 | 1 |
| *Paus ACT15.2* | *Pxyl ACT15.2* | 342 | 287 | 17 | 19 | 0 | 2 |
| *Paus ACT15.3* | *Pxyl ACT15.1* | 350 | 255 | 16 | 21 | 0 | 0 |
| *Paus ACT15.3* | *Pxyl ACT15.2* | 336 | 268 | 10 | 19 | 2 | 2 |
| *Paus ACT15.4* | *Pxyl ACT15.1* | 332 | 268 | 16 | 17 | 0 | 1 |
| *Paus ACT15.4* | *Pxyl ACT15.2* | 321 | 268 | 9 | 21 | 0 | 1 |
| *Paus SA14.1* | *Pxyl SA14.1* | 308 | 249 | 14 | 21 | 2 | 2 |
| *Paus SA14.1* | *Pxyl SA14.2* | 355 | 267 | 20 | 19 | 3 | 2 |
| *Paus SA14.1* | *Pxyl SA14.3* | 353 | 280 | 16 | 15 | 3 | 0 |
| *Paus SA14.1* | *Pxyl SA14.4* | 336 | 284 | 18 | 19 | 0 | 1 |
| *Paus SA14.2* | *Pxyl SA14.1* | 316 | 272 | 22 | 15 | 2 | 1 |
| *Paus SA14.2* | *Pxyl SA14.2* | 338 | 277 | 17 | 18 | 2 | 0 |
| *Paus SA14.2* | *Pxyl SA14.3* | 333 | 272 | 18 | 17 | 2 | 0 |
| *Paus SA14.2* | *Pxyl SA14.4* | 339 | 269 | 25 | 20 | 1 | 2 |
| *Paus SA14.3* | *Pxyl SA14.1* | 338 | 267 | 30 | 21 | 6 | 1 |
| *Paus SA14.3* | *Pxyl SA14.2* | 360 | 302 | 25 | 22 | 5 | 1 |
| *Paus SA14.3* | *Pxyl SA14.3* | 370 | 322 | 17 | 15 | 4 | 0 |
| *Paus SA14.3* | *Pxyl SA14.4* | 353 | 330 | 20 | 27 | 3 | 1 |
| *Paus SA14.4* | *Pxyl SA14.1* | 265 | 238 | 17 | 20 | 2 | 2 |
| *Paus SA14.4* | *Pxyl SA14.2* | 302 | 251 | 18 | 20 | 3 | 4 |
| *Paus SA14.4* | *Pxyl SA14.3* | 290 | 267 | 17 | 14 | 4 | 2 |
| *Paus SA14.4* | *Pxyl SA14.4* | 305 | 270 | 14 | 20 | 2 | 4 |

**Table S7:** Genomic windows with tree-tip distance proportions less than 0.15 below the mean (shortest branch length proportions between a *P. australiana* individual and sympatric Australian *P. xylostella* individual in Figure 5C). The number of times each window appears in a pairwise comparison is indicated (eight pairwise comparisons for population ACT 14, eight for ACT15, and sixteen for SA14).

| Scaffold | Mid Point | Number ACT14 | Number  ACT15 | Number SA14 |
| --- | --- | --- | --- | --- |
| KB207287.1 | 1425000 | 0 | 0 | 1 |
| KB207299.1 | 1475000 | 0 | 0 | 1 |
| KB207303.1 | 525000 | 3 | 0 | 2 |
| KB207304.1 | 1325000 | 0 | 1 | 4 |
| KB207353.1 | 625000 | 2 | 0 | 0 |
| KB207359.1 | 125000 | 3 | 0 | 0 |
| KB207380.1 | 625000 | 6 | 4 | 2 |
| KB207440.1 | 75000 | 1 | 0 | 2 |
| KB207454.1 | 25000 | 0 | 1 | 0 |
| KB207504.1 | 75000 | 3 | 0 | 0 |
| KB207586.1 | 125000 | 0 | 0 | 3 |
| KB207613.1 | 325000 | 0 | 0 | 2 |
| KB207614.1 | 25000 | 0 | 0 | 1 |
| KB207673.1 | 225000 | 0 | 0 | 1 |
| KB207681.1 | 25000 | 0 | 1 | 0 |
| KB207711.1 | 125000 | 0 | 0 | 1 |
| KB207713.1 | 225000 | 0 | 0 | 2 |
| KB207805.1 | 25000 | 0 | 0 | 1 |
| KB207805.1 | 75000 | 1 | 0 | 0 |
| KB207841.1 | 75000 | 0 | 1 | 0 |
| KB207911.1 | 75000 | 2 | 0 | 0 |
| KB207287.1 | 1425000 | 0 | 0 | 1 |
| KB207299.1 | 1475000 | 0 | 0 | 1 |

**Table S8:** Top 1% most divergent windows between *P. australiana* and *P. xylostella* across the genome, determined with *d*_XY_ and F_ST_. (Microsoft Excel spread-sheet).

**Table S9:** Genes models and annotations located within each of the top 1% most divergent windows determined with *d*_XY_ and F_ST_. (Microsoft Excel spread-sheet).
